# Exploring the Role of Birth Environment on Italian Mothers’ Emotional Experience during Childbirth

**DOI:** 10.3390/ijerph20156529

**Published:** 2023-08-05

**Authors:** Laura Migliorini, Nicoletta Setola, Eletta Naldi, Maria Chiara Rompianesi, Laura Iannuzzi, Paola Cardinali

**Affiliations:** 1 Department of Education Sciences, University of Genoa, 16121 Genoa, Italy; 2 Department of Architecture, University of Florence, 50121 Florence, Italy; 3 Provincial Hospital Center AUSL IRCCS Reggio Emilia, 42100 Reggio Emilia, Italy; 4 Centre for Midwifery and Women’s Health, Bournemouth University, Bournemouth BH12 5BB, UK; 5 Department of Economics, Universitas Mercatorum, 00186 Rome, Italy

**Keywords:** birth environment, spatial humanization, affective quality of place, psychological health, women’s perception of birth experience

## Abstract

The physical environment is one of the factors that affect mother’s experience of childbirth and psychological health. A woman’s childbirth experience has been found to influence not only the mother’s own health and future births but also the well-being of her child and family. The present study’s objective was to investigate mothers’ perceptions of spatial-physical humanization, affective quality of place, and emotions during childbirth. To achieve this goal, the first part of our work was dedicated to selecting two birth environments (hospital and birth center) with different degrees of humanization. The methods include observations and field survey which mainly concerned the environmental quality of the spaces and the layout of the birth unit, and self-report questionnaire about perceived environment, affective quality attributed to place, and delivery experience. Participants are 66 low-risk women, choosing hospital or birth center. The findings indicate an enhanced perception of both the spatial-physical aspects and the social and functional aspects of the care unit among mothers who give birth at the birth center. These same mothers also report a more positive perception of the childbirth experience. In conclusion, this study contributes to the understanding of the role of birth environments in shaping mothers’ emotional experiences during childbirth.

## 1. Introduction

Evidence points out that positive relationships with health care providers and good healthcare-built environments play a significant role in patients’ well-being and satisfaction [1]. Several studies from environmental psychology have demonstrated the impact of the spatial and physical conditions of hospital settings on patients’ subjective well-being [2]. Patients in hospitals with better physical conditions have a more positive perceptions of the physical environment and social and organizational relationships [3]. Ulrich’s Theory of Supportive Design [4] provides specific recommendations to reduce stress and enhance well-being. 

Spatial-physical humanization in healthcare places focuses on meeting users’ psychological needs and recognizes the influence of environmental factors on their experiences [5,6]. Although there is increasing attention to designing healthcare facilities to reduce impersonality, few studies include user feedback reliably [7]. Humanization represents a cultural transformation of the care to the users and of the management of work processes, which should pervade all actions and health services. The importance of humanization in healthcare settings has been studied in various contexts, such as pediatric wards and psychiatric emergency units [8,9], underlining the importance of welcoming and humanized care [10]. However, few studies explored the impact of spatial-physical humanization on patients’ perception [11]. International literature established the relation between the design of the hospital setting and the impact on patients, however, there are still few experiences as to birth environment [12]. 

Childbirth is one of the most challenging psychological transitions in a woman’s life, with short- and long-term consequences [13,14]. A recent study highlights the need to identify pathways through research and health care practice that can support women to make informed decisions about pregnancy that resonate with their cultural beliefs as well as the realities of their everyday lives [15].

The “humanization of birth” paradigm prioritizes the interests of mothers and babies, recognizing the influence of birth settings on women’s emotions and birth outcomes. Spatial-physical humanization might influence how women behave, their health and wellbeing, their perception of pain and how they move their bodies [16]. Some studies underline that high women satisfaction was obtained by being in the comfort of their own home and their home environment [17]. Community psychology has recently stressed the importance of psychological home in life transitions as a protection factor for the well-being of people [18]. Hospital birth centers have been associated with lower intervention rates and higher satisfaction [19,20]. The spatial-functional and psycho-sensory characteristics of birth spaces can influence health outcomes and women’s satisfaction [21]. Birth places that do not have a significant domestic identity for women lead them to lose their identity and live the birth passively as a “patient” [22]. Contrary, sufficient space, privacy, soundproofing, and the environment inside the room (adjustable temperature, heating, presence, and spaciousness of private services) represent the key elements for women’s satisfaction [23]. A recent study [24] describes the spatial and physical characteristics related to items of the built environment (e.g., calm atmosphere, greater intimacy, spacious birth room, clarity of service points, clarity in finding midwives, sufficient space for labor, noise, privacy, and the birth room adaptability) experienced as crucial in woman’s experience of birth.

Furthermore, spaces that allow freedom of movement and position changes, and calming birth environment reduce stress [17]. Women who decide to give birth in a birth center are focusing on giving birth in a clean, intimate, and home-like setting [25,26] with the desire to avoid unnecessary medicalization [27]. On the contrary, those who plan on giving birth in hospital value safety and risk perception [28]. The previous study underlines the challenge of creating an optimal birth environment in a hospital setting to satisfy the needs for medico-technical safety, but also physiological and emotional needs of women during labor and birth [29]. Current guidelines for birth space planning and design lack certain characteristics related to humanization that can impact women’s birth experiences [30,31]. When fear of childbirth is exacerbated, it could lead to high stress and anxiety, which can be harmful during pregnancy and childbirth [32]. 

Hofberg and Brockington [33] coined the term tokophobia to refer to a clinically intense fear of childbirth, like a real phobia. Literature underlines that some conditions like unwanted pregnancy, length of labour, and infertility disorders, might be correlated with tokophobia [34].

Traditionally, home was considered the safest place for birth, but in the 1930s, women began moving into institutional birth spaces. Calls for “humanizing birth” led to the development of birth centers, which aim to provide a more homelike environment and pain relief options [35]. Birth centers access is usually strictly limited to women who are at ‘low risk’ of obstetric complications. 

In Italy, maternity care is guaranteed to be free of charge and is part of the National Health System (NHS), which was created in 1978 on the model of the English NHS [36] to ensure universal access to a uniform, free and tax-funded standard of care. The Ministry of Health exercises the function of overall oversight of the health system and sets the essential level of care throughout the country. However, services can also be provided by the private sector within or alongside the SSN. In Italy, women can choose to deliver in hospital, in a midwife-led birth center, which can be located inside or outside the hospital, or at home and in maternity homes [37]. The options depend on women’s needs, risks and choices [19]. Despite the importance of spatial-physical conditions in hospital settings and their impact on well-being, the perception of childbirth places, particularly in Italy, has been underestimated.

In the present study, the initial stage of the methodology involved selecting two birth environments (hospital and birth center) with distinct environmental characteristics, categorized into high and low levels of humanization. The primary focus was to identify environments that varied in terms of their degree of humanization.

The objective of the study was to investigate mothers’ perceptions of spatial-physical humanization, affective quality of place, and emotions during childbirth in these two environments with different environmental features (such as layout, furnishings, and natural light).

## 2. Materials and Methods

The first part of the study consisted in the selection of two birth environments with differing environmental features. Two researchers and practicing architects categorized the birth environments into high and low levels on the degree of humanization using the spatial analysis. It consisted in observations and field survey which mainly concerned the environmental quality of the spaces (furniture, colors, natural light, etc.) and the layout of the birth unit (dimension, flows, spatial relations, etc.). The spatial data collection produced both qualitative evaluation and quantitative measurements.

The second part of the study is related to the measurement of women’s perception of space and emotions because of the experience of childbirth.

After informed consents were obtained, midwives provided a link to fill out the questionnaire on-line, within three months of the birth, to those who have joined the research. This period was chosen because it represents in the literature a particularly delicate moment in which the woman is focused on her child and on the definition of the new identity of mother [38]. In addition, the accuracy of the memory of an event tends to decrease after this time interval [39].

The study was conducted after obtaining the approval of both the local hospital Ethics Committees (Auslre, n. 2018/0058874; Regional Ethical Committee for Clinical Trials of the Tuscany Region n. 12366_oss). The data collection procedure was in line with the Research Ethical Code of the Italian Association of Psychology and the ethical recommendations of the Declaration of Helsinki, as well as the American Psychological Association (APA) standards for the treatment of human volunteers.

### 2.1. Participants

In total, 100 women were invited from midwife, but only 66 mothers, 30 of whom were planning birth in hospital, and 36 in a birth center, accepted and completed the study. They have a mean age of 35.1 years. We select only low-risk women, whose childbirth was identified as straightforward according to local protocols of the chosen facility (hospital or birth center). Women who experienced induction, augmentation, instrumental assistance, caesarean section, epidural anesthesia or other pain relief medications were excluded. Exclusion criteria have been also considered: the lack of knowledge of the Italian language, the lack of access to means to compile online questionnaires, under 18 years of age or over 43 years, within the physiological range according to healthcare protocols for childbirth.

The study was carried out in two birthplaces located in central Italy in the period between June 2018 and December 2019.

### 2.2. Data Collection Tools

This research used the online survey method for quantitative data collection.

The self-report questionnaire comprised several measures:

Socio-demographic information. Respondents were asked to provide some socio-demographic data (age, primiparity, gestational week at delivery, educational level, marital status).

Perceived Hospital Environment Quality Indicators (PHEQIs) (labeled in Italian “Indicatori di Umanizzazione Ospedaliera Percepita”—IUOP—brief version) [10]. The scale is made up of 25 items with a 5-point Likert response scale, from 1 = completely in disagreement to 5 = completely in agreement. The scale consists of two dimensions: physical-spatial indicators (building aesthetic, acoustic comfort, climate comfort, care for waiting or meeting rooms, views, orientation, and reception); socio-organizational indicators (organizational clarity, privacy, availability, and professionalism of operators).

Scales of the Affective Quality attributed to Place (Q.A.L.) [40], Italian version [41]. Q.A.L. is a self-report questionnaire, consisting of 48 items divided into 4 sub-scales that represent bipolar and orthogonal axes. Each axis is composed of two opposite dimensions: relaxing vs. stressful and exciting vs. depressing; pleasant vs. unpleasant and stimulating vs. soporific/boring. Each pole has 6 qualifying adjectives. Each adjective is accompanied in the questionnaire by a scale of evaluation to indicate the extent to which the adjective is, in the opinion of the subject, “suitable to affectively describe” the place under consideration. Adjectives are evaluated on a Likert scale of 7 points: from 0 (not at all suitable) to 6 (completely suitable). The total score for each sub-scale is calculated by adding the attribute value from the subject to each of the 6 adjectives that make up that size and can vary from a minimum of 0 to a maximum of 36. If the subject totals in a certain dimension, for example in Relaxing sub-scale, a score of 0 or 36 indicates, respectively, that the above dimension is not suitable or is entirely suitable to describe his affective perception of the environment.

Wijma Delivery Experience Questionnaire (WDEQ-version B) [42] is a self-report questionnaire aimed at measuring the emotions associated with childbirth, after the birth event. It consists of 33 items, which saturate six factors: Factors included lack of self-efficacy, loneliness, negative appraisal, lack of positive anticipation, fear, and concern for the child. Answers are given on a 6-point scale ranging from ‘not at all’ (0) to ‘extremely’ (5), yielding a maximum score of 165 and a minimum score of 0. A higher score indicates a more severe fear of childbirth. W-DEQ score of ≥85 is considered to indicate severe fear of childbirth.

Completing the questionnaire took a mean of 20 min.

### 2.3. Data Analysis

#### 2.3.1. Spatial Analysis

The spatial analysis includes various qualitative and quantitative techniques to study the environmental property of the birth spaces. Firstly, the researchers conducted field research of the two settings consisting of collecting data by direct observations (site survey) of the built environment and taking pictures of the spaces according to the users’ activities. In addition, the floorplan of the two settings permitted to broaden the field survey with measurements of the spaces and spatial layout considerations (flows of stakeholders, relations between the spaces, etc.). using tools such as Space Syntax. Both methods are fully explained in the previous article on the same research [24]. The data were collected and evaluated following the scientific literature on the built environment’s effect on maternity outcomes [43]. Particular attention was paid to the spatial-physical humanization factors of the perceived quality of the environment indicated in the Questionnaire.

#### 2.3.2. Statistical Analysis

We used Jamovi Statistical Software for Social Science to compute scores on the variables of interest, perform descriptive analyses, and calculate the Cronbach’s alpha. 

First, Pearson’s Chi Square analyses were conducted to investigate the homogeneity of the two conditions relating to the main demographic variables. 

We then compared the two conditions with Student’s *t*-test to assess the presence of differences in the perception of spatial-physical humanization, affective quality of place, and emotions during childbirth between women who gave birth in a hospital and those who gave birth in a birth center. 

For all inferential tests, a *p*-value of 0.05 or less was considered significant given the small sample size of this exploratory study.

## 3. Results

### 3.1. Identifying the Study Sites

The study was carried out in two birthplaces in two Italian teaching hospitals with significantly different spatial, physical, and social and functional aspects of the environment, reflecting a diverse level of spatial-physical humanization.

The different design assumptions and aims of care of the two places are already revealed to layout level with substantial differences: the hospital has a typical triple-body configuration in which the rooms follow one another linearly; the Birth Center is an independent building and entirely dedicated to the physiological path, the layout gives the spaces a more intimate and collected conformation, with the central obstetric desk and the rooms that develop concentrically around it. These settings, with their deliberately dissimilar peculiarities, have been identified as ideal for describing settings with high and low levels on the degree of spatial-physical humanization and for exploring how environmental factors can determine similarities and differences in the affective perception of a place of birth by women. 

The Hospital setting is representative of a typical Italian labor and maternity ward, whereas the Birth Center Unit was selected between three Italian-recognized Birth Centers for its innovativeness; it is the only one designed and realized specifically as Birth Center.

Case 1—Hospital setting (low degree of spatial-physical humanization setting)

**Figure 1:**
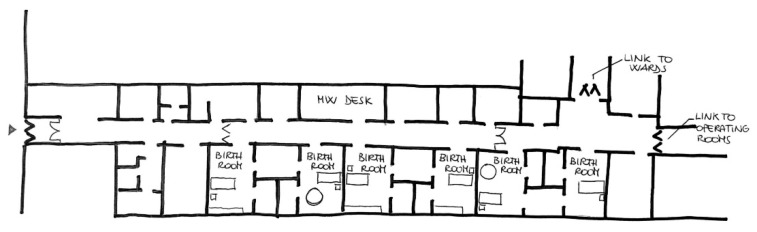
Architectural plan of the Hospital setting.

The Case 1 setting provides care to high and low-risk women thanks to a team of midwives and doctors. In this setting, mothers don’t spend their entire stay in the hospital’s Labor, Delivery, and Recovery (LDR) rooms, because they are moved into a separate postpartum area following the birth. The labor and birth ward, situated on the hospital’s second floor, is a typical hospital ward, with pillars imposing a rectangular layout where the rooms overlook a long corridor where the main flows are concentrated (Figure 1). The standardized load-bearing structure imposes a rigid position on the different areas, with no pleasant view outside the main corridor and the presence of typical hospital equipment around. In the hospital there are no waiting areas for partners or supporters who have only the possibility to enter the LDR room with only a chair to rest, and any food and drink at their disposal inside the Unit.

The six rectangular LDR rooms measure around 25 m^2^ comprehending the en-suite bathroom. A resuscitation room is in common for every two rooms. The rooms, quite spacious, have as the focus the bed, around which all the equipment is organized, except for those two which have a birthing pool inside. The rooms are bright thanks to a big window overlooking the outside (the view is facing another brick building of the hospital, so not to nature and not particularly restorative), and the warm colors of the cream-colored floor and walls, white and pale yellow, with orange doors. The feeling provided by the room is to be in a hospital due to the medical equipment on sight, the hospital bed, and the type of furnishing. The sense of privacy is not highly guaranteed since the door opens directly to the main corridor where all the main activities happen, and the users walk.

Case 2—Birth Centre setting (high degree of spatial-physical humanization setting)

The Case 2 setting, the alongside midwife-led unit, run by a team of midwives, provides care to women with straightforward pregnancies who decide to give birth physiologically. In this setting, mothers usually spend their entire stay since the midwives are responsible for ante, intra, and postpartum care: after birth, they have the possibility to stay in their Labor, Delivery, Recovery, and Postpartum (LDRP) rooms for the other 48 h. 

**Figure 2:**
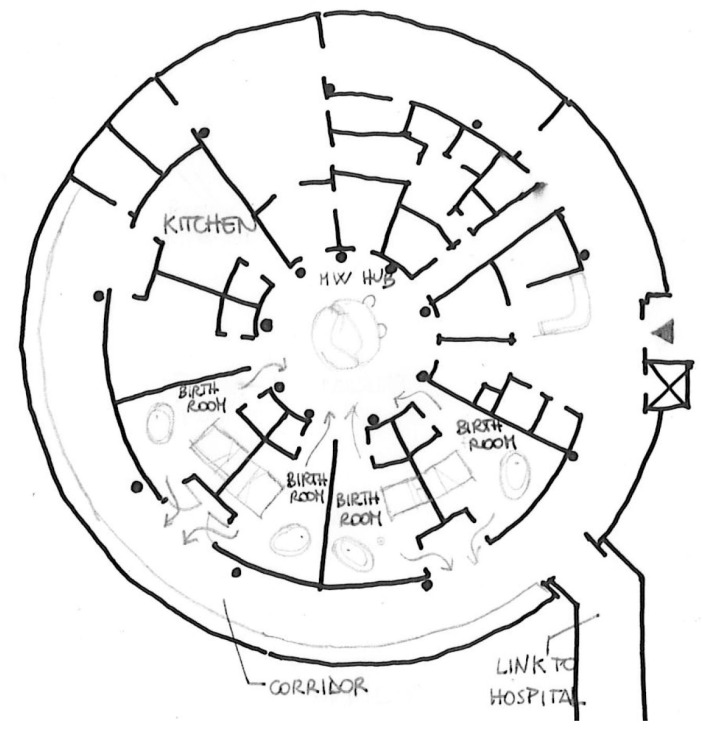
Architectural plan of the Birth Centre setting.

The Unit is located on the first floor of a two-floor independent building that has a round plan and thanks to a short corridor is directly linked to another building dedicated to Maternity care, so in case of need or emergency women and/or babies can be rapidly connected to the Obstetric-Led Unit, Neonatal Unit and Caesarean Section theater. The layout of the Unit is unusual for a healthcare setting: it is based on a circular system organized radially around a central space, where the midwives’ workstation is located (Figure 2). Around this core, all the functional spaces are organized, among them five LDRP rooms, and a common kitchen for visitors and staff members. The third and outer ring of the layout is a wide, bright corridor with large windows overlooking the landscape outside. This hosts the flow for visitors who can enter the rooms directly from a secondary entrance and a social space where they can wait, walk, and exchange experiences. The five birth rooms measure around 34 m^2^ comprehending the en-suite bathroom (without shower) and present as the focus of the room a colored birthing pool, followed by a home-like double bed and various furnishings that foster physiologic birth and emergency equipment hidden within the furniture. The LDRP rooms are not particularly bright because the windows are positioned at the top of the wall, while the central area housing the midwives’ desk and the corridor outside are bright; the latter also enjoys a view of the green. The atmosphere is welcoming and domestic due to the particular furniture. In addition, medical equipment is present but hidden within the furniture. The sense of privacy is guaranteed as the LDRP room has two access possibilities: a more lateral one that faces the corridor and can be closed if necessary, and a more central one that connects directly with the midwives’ desk.

### 3.2. Mothers’ Perceptions

The mothers included in the study have an average age of 34.13 years in the hospital context and of 33.94 years for those who give birth at the birth center. We have a 53.3% primiparous in the hospital context, while 50% of those who are admitted to the Birth Center declare to be at the first pregnancy. In both contexts almost all of the participants declare themselves married (96.7% hospital context; 94.4% birth center). Thirty percent of participants have a university degree in hospital (33.3%) and in birth center (30.6%) and in most of the respondents (80% in hospital; 88.9% in birth center) were employed, followed by a small group of housewives (6.7% in hospital; 2.8% in birth center). Unemployed people represented 5.6% of the total respondents in birth center. Chi-Square analysis revealed that the two groups are homogenous in all demographics.

Statistical processing produced a total of 9 indicators of Perceived Hospital Environment Quality [10]. Most dimensions contained both positive (presence of quality) and negative (lack of quality) items. In computing the scores, negatively worded items have been reversed so that high factor scores would always refer to a positive evaluation.

As presented in Table 1 the first six dimension refers to spatial physical aspects of the care unit, while the last three are related to Social and functional aspects of the care unit.

All dimensions exhibit excellent internal consistency with Cronbach’s Alpha above 0.80, except for the View dimension, which has an Alpha above 0.60, and the Privacy dimension, which has a Cronbach’s alpha of 0.45.

In the hospital context, the highest averages are related to Privacy (m = 4.52) and Building aesthetics (m = 4.04), while the least humanized dimensions result in Views (m = 2.22) and Care for waiting or meeting rooms (m = 2.37). 

In the Birth Center, the highest averages are relative to Privacy (m = 4.81) and Availability and Professionalism of operators (m = 4.80), while the dimension less humanized turns out to be Views (m = 2.66) and Climatic comfort (m = 3.65). 

The Student T test for independent samples was conducted on the two groups (Table 1) and showed a statistically significant difference between the two groups in all indicators except for Climate Comfort and Views.

**Figure 3:**
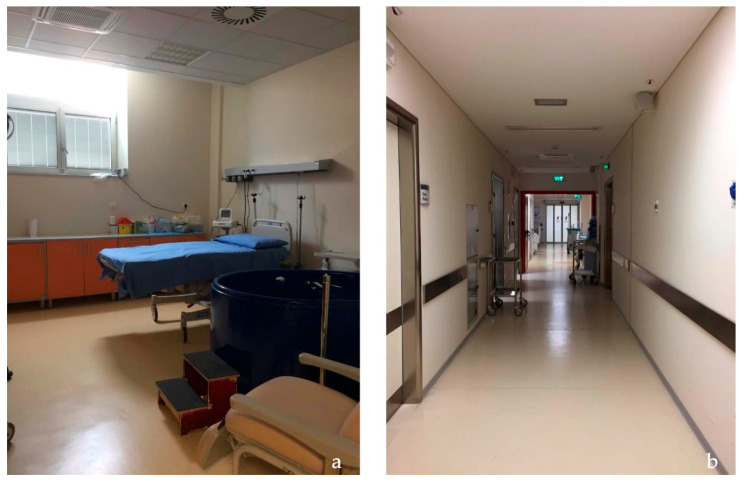
Birth room (**a**) and corridor (**b**) of the Hospital.

**Figure 4:**
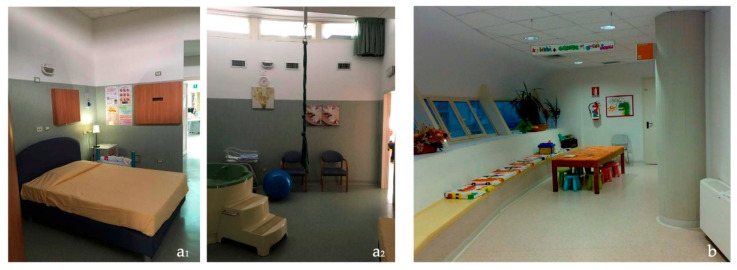
Birth room (**a1**,**a2**) and corridor (**b**) of the Birth Centre.

Building aesthetics have a more positive assessment in the Birth Centre setting (t(64) = 3.35, *p* < 0.005). Spatial analysis has shown that the condition and quality of furnishing in Hospital are good, and coherent with a hospitalized setting hosting recent equipment and furnishing; walls, floors, and ceilings are well maintained and clean (Figure 3). However, in the Birth Center, there is more domestic furniture, thanks to the presence of a non-hospital double bed, the use of wood, and the colors characterizing each room’s walls and floors (Figure 4).

In the dimension Acoustic comfort the *t*-test value was t(64) = 3.09 with a *p*-value of *p* < 0.005, indicating a statistically significant difference in the perception, with a higher mean in the Birth Center. Direct observations of the built environment reveal that inside the Hospital, acoustic comfort is not completely guaranteed in the rooms because it may happen that the quietness is disturbed by the noise coming from the outside, being the rooms facing the unique long corridors where all the flaws pass through. On the contrary, in the Birth Center, the layout promotes better acoustic comfort because the presence of two doors permits better screening of the noise and creates a calmer working station to which doors open.

Perception about care for waiting or meeting rooms is significantly lower in Hospital (t(64) = 9.65, *p* < 0.000), this is coherent with spatial analysis that highlights that they are insufficient for the purposes of the Unit. The staff has a little space to rest and eat but could be better equipped both as a kitchen and as a resting area; external visitors don’t have waiting or lounge rooms, so they are forced to wait/have a snack outside the unit or inside the hospital rooms where no specific space is dedicated to them. In the birth center, there are various waiting areas for partners or supporters, who have the possibility to wait and socialize in the outer corridor where seats are specifically located for them, including an area for kids, or use the little kitchen or enter the LDRP rooms where is also offered the possibility to spend the night in the double bed. 

Regarding orientation and reception, the significant difference in women’s perception revealed from the questionnaire (t(64) = 3.74, *p* < 0.000) is in line with spatial observations that reveal that in the hospital the orientation is great, the layout has a functional and standard design that creates simple wayfinding, allowing visitors to presume the logic in orientation, but the highly repetitive and squared spaces don’t easily indicate the position of information points, only insufficient signposting allows them to find what they are looking for (Figure 1). The unit’s entrance is not particularly welcoming, whit a hospital-like door closed to impede the entrance. On the contrary, the ‘unusual’ layout of the birth center with a strong centrality of the midwives’ position, allow mothers to find what they are looking for. The unit’s entrance is not particularly recognizable or welcoming, but the doors open to the inner circle facilitate the possibility to see or find the staff.

The spatial organization of the hospital unit, rigid and standardized, is not immediately reflected in an organization clarity where patients can identify the name and function of the staff members or whom to ask for information, in fact, the staff workstation is not on sight and is not clear where they could be or where to wait for them, adding the fact that other healthcare professionals may pass through the corridor, directed in another care unit. The *t*-test shows a significant difference between the two groups in the perception of organization clarity (t(64) = 4.76, *p* < 0.000). 

Privacy has high scores in both settings, however, there is a significantly more positive perception in the birth center (t(64) = 2.11, *p* < 0.05). In the hospital layout, the unit imposes that the door of the LDR opens to the corridor where could pass staff members, visitors, or other pregnant women, but can prevent the sensation of mothers being watched since doors are usually closed and the unit is not often too crowded. In the birth center, it is possible to notice that mothers may perceive a higher sense of privacy thanks to the fact that the setting is generally less crowded than the unit because there are no staff or visitors passing through, and that the two doors prevent the sensations of being watched from ‘strangers’ since the inner door, used by midwives and likely more open, face to a working area, where there are usually no visitors.

Finally, the dimension Availability and professionalism of operators has been evaluated more positively by mothers that gave birth in the birth center (t(64) = 4.85, *p* < 0.000). In the hospital the availability of operators is not favored by the configuration of the Unit because the staff members don’t have complete control of the visitor flow, since their workstation is located in those rooms on side of the long corridor, so they are not facilitated in providing a nice welcoming to visitors or create a good cooperative atmosphere with visitors since they don’t share a pleasant or relaxing space in which to share comments or have a conversation. On the other hand, the spatial analysis conducted on the birth center reveals that staff is supported by the configuration of the unit because they operate in the more integrated area of the unit; from the working station midwives easily control the birth rooms and the entrance flows which facilitate a nice welcome. In addition, a shared kitchen, and a restroom next to a unique working station favor a good cooperative atmosphere among staff members which has easier and more immediate communication thanks to the layout.

In two dimensions there are no differences in the perception of the two groups: climate comfort and View. This appears quite in relation to the spatial characteristics detected by the observational measurements of the two settings. In fact, good climate comfort is guaranteed in both settings (although not adjustable), even if the big windows of the hospital unit may permit a greater air exchange from the outside and suitable air humidity in all the rooms, except for the long corridor. Concerning the views, the windows of the LDR rooms provide views of the outside; green spaces are not visible, but the view has anyway little interest, especially for the sunlight provided. Conversely, the birth room of the second setting doesn’t allow any view to the outside, except those permitted to the skylight, because the windows are at a high level, not at eye level, so it is not possible to benefit from the view of green spaces which is instead possible from the big windows of the external corridor. 

In both groups, social and functional indicators achieve higher scores than spatial physical ones, highlighting a greater impact of these elements on the perception of spatial-physical humanization of the context.

**Figure 5:**
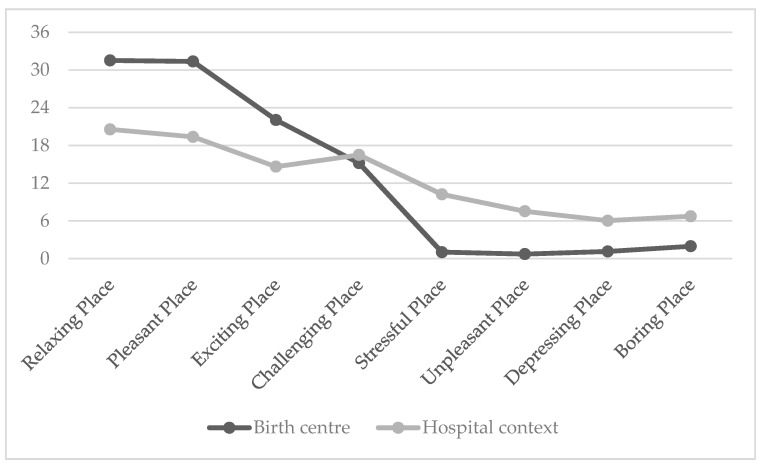
Mean scores in Affective Quality of Place in hospital and birth center.

As regards the affective quality of the place (Figure 5) for the adjective Relaxing the mean score was 31.53 (SD = 3.99) in the birth center and 20.57 (SD = 8.21) in the hospital context. The *t*-test yielded a significant difference (t(64) = 7.07, *p* < 0.001) between the two settings, indicating that the hospital context elicited significantly lower perceptions of relaxation compared to the birth centre. Regarding the dimension Pleasant, the mean score was 31.36 (SD = 4.45) in the birth centre and 19.37 (SD = 8.67) in the hospital context. The t-test showed a highly significant difference (t(64) = 7.24, *p* < 0.001) between the two settings, suggesting that the birth centre was perceived as significantly more pleasant than the hospital context. For the emotional descriptor Exciting, the mean score was 22.05 (SD = 7.57) in the birth centre and 14.63 (SD = 9.34) in the hospital context. The *t*-test revealed a significant difference (t(64) = 3.49, *p* = 0.001) between the two settings, indicating that the birth centre was associated with higher perceptions of excitement compared to the hospital context. Regarding the emotional characteristic Challenging, the mean score was 15.19 (SD = 5.53) in the birth centre and 16.50 (SD = 6.81656) in the hospital context. The *t*-test did not yield a statistically significant difference (t(64) = 0.403, *p* > 0.05) between the two settings for this affective quality of the place.

For the adjective Stressful the mean score was 1.03 (SD = 1.83) in the birth centre and 10.23 (SD = 8.25) in the hospital context. The *t*-test showed a highly significant difference (t(64) = −6.52, *p* < 0.001) between the two settings, indicating that the hospital context was perceived as significantly more stressful than the birth centre. Regarding the emotion label Unpleasant, the mean score was 0.72 (SD = 1.36) in the birth centre and 7.53 (SD = 7.37) in the hospital context. The *t*-test revealed a highly significant difference (t(64) = −5.438, *p* < 0.001) between the two settings, suggesting that the hospital context was associated with significantly higher perceptions of unpleasantness compared to the birth centre. For the emotional descriptor Depressing, the mean score was 1.14 (SD = 1.89) in the birth centre and 6.03 (SD = 7.07) in the hospital context. Also for this adjective the *t*-test showed a highly significant difference (t(64) = −3.988, *p* < 0.001) between the two settings, indicating that the hospital context elicited significantly higher perceptions of depression compared to the birth centre. Finally, for the adjective Boring, the mean score was 1.97 (SD = 4.07) in the birth centre and 6.73 (SD = 7.02) in the hospital context. The *t*-test revealed a significant difference (t(64) = −3.439, *p* = 0.001) between the two settings, suggesting that the hospital context was perceived as significantly more boring than the birth center. 

Compared to the overall score of the Wijma Delivery Experience Questionnaire scale, there is a statistically significant difference between the two contexts (t(64) = −4.62; *p* < 0.001). In addition, the average score in the hospital context was 96.17 (SD = 16.29) and exceeds the threshold value of 85, considered critical in the literature, while in the Birth Center the average score is 74.89 (SD = 20.34). 

In Table 2 we present Alpha di Chronbach of the different subdimension of Wijma Delivery Experience Questionnaire. All dimensions exhibit good internal consistency with Cronbach’s Alpha above 0.60, except for Lack of positive anticipation which has a Cronbach’s alpha of 0.45.

As presented in Table 2 for the subdimension Low Self-Efficacy, the mean score was 25.44 (SD = 7.34) in the birth center and 31.17 (SD = 5.97) in the hospital context. The *t*-test revealed a significant difference (t(64) = −3.50, *p* < 0.001) between the two settings, indicating that the hospital context was associated with significantly higher perceptions of a lack of self-efficacy compared to the birth center. Regarding the subdimension Loneliness, the mean score was 15.22 (SD = 5.98) in the birth center and 21.53 (SD = 5.21) in the hospital context. The *t*-test yielded a highly significant difference (t(64) = −4.52, *p* < 0.001) between the two settings, suggesting that the hospital context elicited significantly higher feelings of loneliness compared to the birth center. For the Negative Appraisal the mean score was 10.56 (SD = 4.10) in the birth center and 12.17 (SD = 3.51) in the hospital context. The *t*-test did not show a statistically significant difference (t(64) = −1.99, *p* = 0.095), between the two settings for this subdimension. Regarding the Lack of Positive Anticipation, the mean score was 8.25 (SD = 3.07) in the birth center and 10.83 (SD = 3.14) in the hospital context. The *t*-test revealed a significant difference (t(64) = −3.37, *p* = 0.001) between the two settings, indicating that the hospital context was associated with significantly lower levels of positive expectations compared to the birth center. For the dimension Fear, the mean score was 12.42 (SD = 3.74) in the birth center and 16.10 (SD = 3.56) in the hospital context. The *t*-test showed a highly significant difference (t(64) = −4.07, *p* < 0.001) between the two settings, suggesting that the hospital context was associated with significantly higher levels of fear compared to the birth center. Finally, regarding the Concern for the Baby, the mean score was 3.00 (SD = 1.60) in the birth center and 4.37 (SD = 2.30) in the hospital context. The *t*-test revealed a significant difference (t(64) = −2.84, *p* = 0.006) between the two settings, indicating that the hospital context was associated with significantly higher levels of concern for the baby compared to the birth center.

Overall, the findings indicate that for the subdimensions of the delivery experience, women in birth center showed significantly lower means in Low self-efficacy, Loneliness, Negative appraisal, Lack of positive anticipation, Fear, and concern for the baby compared with women in hospital.

## 4. Discussion

Researchers know that childbirth represents a major life event in women and family life. Childbirth experiences are subjective psychological and physiological processes, influenced by social and environmental factors. In the context of childbirth, spatial-physical humanization involves creating a comfortable and supportive environment that empowers women, increasing the wellbeing. However, literature highlights the limited knowledge on how birth environments may affect the health of birthing women [44] and the limited listening to women’s lived experiences, often seen only as consumers of these units [29]. 

The objective of the current research was to contribute to reflection on birth environmental role on mother emotional experience of childbirth. In the first part of the study, hospital and birth center, identified as ideal for exploring how environmental factors can determine similarities and differences in the affective perception of a place of birth by women, were analyzed and categorized into high and low levels on the degree of humanization using the spatial analysis. The presentation of the two case studies underlines how different spatial, physical design, and social and functional attributes are associated to different levels of spatial-physical humanization and their relationship with wellbeing. In this regard, recently, Cho [45] investigated architectural designers’ point of view of the importance of healthcare environmental criteria in the implementation of user-centered, therapeutic hospital design, discussing the barriers and suggestions for the healthcare design practice. The environmental comfort quality criterion (i.e., illumination, climate comfort) has received attention in some publications, as environmental attributes affect hospital users’ healing and well-being [46]. In the present study there are several dimensions refer to spatial physical aspects (e.g., building aesthetic, acoustic and climate comfort) of the care unit that have higher scores in the group of mothers who experience childbirth in the birth center than in those who experience childbirth in the hospital context. Furthermore, in line with a review [47], service environment attributes, including supportive service from healthcare workers (availability and professionalism of operators) and family service-related physical facilities (e.g., care for waiting room), are better perceived in birth center than in hospital.

Present study findings support the hypothesis that the different degree of objective physical-spatial humanization of the environment dedicated to labor and childbirth is reflected in the perception of users, both regarding the physical-spatial, and socio-organizational aspects that characterize the setting. From this comparison it emerges, in line with the studies of Fourier and Herter [35], how the birth center represents a more human environment, which fits with the needs of mothers and family members at birth.

Both settings are perceived with a positive affective quality, the “relaxing” and “pleasant” dimensions have higher scores than the opposite “stressful” and “unpleasant”; however, the birth center is perceived significantly more positively from an affective point of view by mothers. Specific locations and settings, like the childbirth setting, may acquire some form of special meaning for people. Affective evaluations could be very much ‘place centered’ [48] and could be the result of relations between the mothers, the setting, and other participants within that setting. Positive bonds are generally developed when the place provides people with security, and familiarity, and facilitates functioning [49]. In line with this research our study underlines the importance of the patient’s perception of hospital facilities especially in challenging.

In a previous work [24] some authors find that calm environment and flexible and intelligible environment summarize the characteristics that all birth environments should have to contribute to the physiological process of birth and consequently, to a better childbirth experience. 

Regarding the delivery experience, mothers who give birth in the birth center perceive the experience less negatively than those who give birth in the hospital in terms of fear, loneliness, low self-esteem, concern for the child. This work represents a first exploration of the importance of physical-spatial humanization in promoting well-being during a delicate time in the life of women and the family as childbirth. By prioritizing the individual’s physical and emotional well-being, spatial-physical humanization can help create a safe and supportive environment for delivery. Future studies could explore how spatial-physical humanization can promote a sense of trust and connection between the woman and care providers, and how this can further reduce fear and anxiety. 

Recent work analyzes the impact of differently designed birthing rooms at a hospital-based labor ward [29], our study expanded this observation to the entire unit design. Furthermore, our work is the first in the Italian context to analyze women’s perceptions of the perceived humanization and affective quality attributed to places of birth. The integration between the observations of researchers and practicing architects, on the one hand, and the perceptions of mothers, on the other, is an interesting aspect of the present study. 

## 5. Limitations and Future Research

There are several limitations in this study. First of all, the small number of participants does not allow us to generalize our observations to other contexts, however it is important to remember that childbirth, and the period after childbirth, are delicate moments in the life of the woman and the family and that it can therefore be very difficult to reach the participants. Secondly, due to the correlative nature of the research design, it is not possible to prove the existence of causal relationships between perceived spatial-physical humanization, affective quality of place and mothers’ emotions towards birth.

Third, our results on the better perception of childbirth event in BC than in hospital can be considered associated with the mothers’ choice to give birth in this peculiar space [50]. This choice can play an important role in the perception of the affective quality of the place, of the relationship with the healthcare personnel, and in the perception of the birth event. Further research, with greater sampling size, could be directed to the analysis of the antecedents of this choice, and to their role in mediating the relationship between objective spatial features and perception. The inclusion of other points of view, such as those of partners or midwives, could also provide a more complex and clearer picture on this issue. Furthermore, the reliability of two dimensions in two different scales remains uncertain as their Cronbach’s alpha falls within the range of 0.40 to 0.60, suggesting that caution should be exercised when interpreting the findings related to these dimensions.

Finally, it is important to note that the data were collected before the COVID-19 Pandemic, which had a significant impact both on the well-being of new mothers [51] and on the organization of spaces as a result of the security measures introduced in hospitals [52]. Future research could highlight the importance of some specific elements of spatial organization that have so far been taken for granted.

## 6. Conclusions

The study broadens the knowledge about the mothers’ perception of the birth physical environment and develops maternity spaces design knowledge. The findings highlight the importance of spatial-physical humanization in creating a supportive and empowering environment for women. The birth center, characterized by higher levels of spatial-physical humanization, was perceived more positively by mothers compared to the hospital setting. These perceptions were reflected in affective evaluations, with the birth center being associated with feelings of relaxation and pleasure. Furthermore, mothers who gave birth in the birth center reported a less negative delivery experience in terms of fear, loneliness, low self-esteem, and concern for the child. The study underscores the importance of considering the subjective experiences and perceptions of women when designing birth environments. Affective evaluations of the environment play a crucial role in shaping the overall childbirth experience. Our study gives valuable insight into environmental variables associated with women emotions in childbirth experience thanks to the encounter between the disciplines of architecture and design and environmental and perinatal psychology. Multidisciplinary has made it possible to integrate different perspectives that allow a better understanding of the relationship between spatial elements and emotional well-being. Furthermore, our study could have implications for the design of the birth environments and health-care policy, supporting birth centers. In conclusion, by prioritizing physical and emotional well-being, spatial-physical humanization can contribute to a better childbirth experience.

## Figures and Tables

**Table 1:** Independent Sample *t*-test related to Perceived Hospital Environment Quality Indicators among Hospital and Birth Center.

Indicators	Setting	N	Mean	SD	Student’s *t*	Sig.
Building aesthetics (Alpha 0.88)	Hospital	30	4.04	0.80		
	BC	36	4.62	0.59	3.35	0.001
Acustic comfort (Alpha 0.82)	Hospital	30	3.75	1.27		
	BC	36	4.55	0.71	3.09	0.003
Climate comfort (Alpha 0.83)	Hospital	30	3.52	1.07		
	BC	36	3.65	1.09	0.51	0.612
Care for waiting or meeting rooms (Alpha 0.86)	Hospital	30	2.38	0.88		
	BC	36	4.38	0.80	9.65	0.000
Views (Alpha 0.69)	Hospital	30	2.22	0.68		
	BC	36	2.67	1.22	1.86	0.068
Orientation and reception (Alpha 0.87)	Hospital	30	3.40	1.11		
	BC	36	4.30	0.80	3.74	0.000
Organisation clarity (Alpha 0.88)	Hospital	30	3.48	1.06		
	BC	36	4.54	0.66	4.76	0.000
Privacy (Alpha 0.45)	Hospital	30	4.52	0.67		
	BC	36	4.81	0.39	2.11	0.041
Availability and professionalism of operators (Alpha 0.85)	Hospital	30	3.91	0.67		
	BC	36	4.80	0.32	4.85	0.000
Spatial physical aspects of the care unit (includes first six dimensions)	Hospital	30	3.22	0.57		
	BC	36	4.03	0.60	5.55	0.000
Social and functional aspects of the care unit (includes last three dimensions)	Hospital	30	3.97	0.74		
	BC	36	4.72	0.35185	5.05	0.000

**Table 2:** Independent Sample *t*-test related to Delivery Experience among Hospital and Birth Center.

	Group	N	Mean	SD	Student’s *t*	*p*
Low Self efficacy (Alpha 0.86)	Hospital	30	31.17	5.97	−3.50	0.001
	Birth Center	36	25.44	7.34		
Loneliness (Alpha 0.79)	Hospital	30	21.53	5.21	−4.52	<0.001
	Birth Center	36	15.22	5.98		
Negative appraisal (Alpha 0.72)	Hospital	30	12.17	3.51	−1.99	0.095
	Birth Center	36	10.56	4.10		
Lack of positive anticipation (Alpha 0.45)	Hospital	30	10.83	3.14	−3.37	0.001
	Birth Center	36	8.25	3.07		
Fear (Alpha 0.67)	Hospital	30	16.10	3.56	−4.07	<0.001
	Birth Center	36	12.42	3.74		
Concern for the baby (Alpha 0.72)	Hospital	30	4.37	2.30	−2.84	0.006
	Birth Center	36	3.00	1.60		

## Data Availability

The data for this research study will be made available upon request to authorized researchers and will be stored securely in accordance with data protection regulations.
